# Extracellular Vesicles from Hyperammonemic Rats Induce Neuroinflammation and Motor Incoordination in Control Rats

**DOI:** 10.3390/cells9030572

**Published:** 2020-02-28

**Authors:** Paula Izquierdo-Altarejos, Andrea Cabrera-Pastor, Hernan Gonzalez-King, Carmina Montoliu, Vicente Felipo

**Affiliations:** 1 Laboratory of Neurobiology, Príncipe Felipe Research Center, 46012 Valencia, Spain; pizquierdo@cipf.es; 2 Health Research Institute INCLIVA, 46010 Valencia, Spain; acabrera@cipf.es (A.C.-P.); cmontoliu@incliva.es (C.M.); 3 Regenerative Medicine and Heart Transplantation Unit, Health Research Institute La Fe, 46026 Valencia, Spain; hernangkg10@gmail.com; 4 Pathology Department, University of Valencia, 46010 Valencia, Spain

**Keywords:** hepatic encephalopathy, TNFα, TNFα receptor TNFR1, glial activation

## Abstract

Minimal hepatic encephalopathy is associated with changes in the peripheral immune system which are transferred to the brain, leading to neuroinflammation and thus to cognitive and motor impairment. Mechanisms by which changes in the immune system induce cerebral alterations remain unclear. Extracellular vesicles (EVs) seem to play a role in this process in certain pathologies. The aim of this work was to assess whether EVs play a role in the induction of neuroinflammation in cerebellum and motor incoordination by chronic hyperammonemia. We characterized the differences in protein cargo of EVs from plasma of hyperammonemic and control rats by proteomics and Western blot. We assessed whether injection of EVs from hyperammonemic to normal rats induces changes in neuroinflammation in cerebellum and motor incoordination similar to those exhibited by hyperammonemic rats. We found that hyperammonemia increases EVs amount and alters their protein cargo. Differentially expressed proteins are mainly associated with immune system processes. Injected EVs enter Purkinje neurons and microglia. Injection of EVs from hyperammonemic, but not from control rats, induces motor incoordination, which is mediated by neuroinflammation, microglia and astrocytes activation and increased IL-1β, TNFα, its receptor TNFR1, NF-κB in microglia, glutaminase I, and GAT3 in cerebellum. Plasma EVs from hyperammonemic rats carry molecules necessary and sufficient to trigger neuroinflammation in cerebellum and the mechanisms leading to motor incoordination.

## 1. Introduction

Patients with liver cirrhosis may present minimal or clinical hepatic encephalopathy (HE), a complex neuropsychiatric syndrome leading to cognitive and motor alterations which reduces quality of life and life span of the patients. Hyperammonemia and inflammation act synergistically to induce minimal HE (MHE) [1,2,3,4,5].

Studies in patients and in animal models show that MHE appearance is associated with changes in the peripheral immune system that are transferred to the brain, inducing neuroinflammation, which in turn leads to cognitive and motor impairment [6,7,8,9,10,11].

Changes in the immune system also lead to cerebral alterations in pathologies associated to sustained peripheral inflammation such as diabetes, rheumatoid arthritis, obesity, and chronic kidney disease, as well as neurological and neurodegenerative diseases such as schizophrenia, Parkinson’s and Alzheimer’s disease [12,13,14,15,16,17,18,19,20].

The mechanisms by which changes in the immune system induce alterations in brain in response to sustained inflammation are not well understood. There is increasing evidence that extracellular vesicles (EVs) may play an important role in this process [21]. EVs are membranous vesicles released by most cells [22]. EVs play a main role in the mediation of immune and inflammatory responses, and in diseases with a significant inflammatory component [23,24].

EVs may act as mediators of neuroinflammation [25] and may transmit pathological effects from the periphery to the brain. For example, EVs are increased in serum of children with autism spectrum disorder and stimulate human microglia to secrete IL-1β, which may explain what triggers inflammation in brain of these children [26].

EVs of sporadic amyotrophic lateral sclerosis (ALS) patients transport pathological proteins which might play a role in prion-like propagation of ALS disease [27].

Han et al. [28] showed that intravenous treatment of mice with exosomes from serum of patients with Parkinson´s disease evoke protein aggregation, trigger dopamine neuron degeneration, induce microglial activation, and cause movement defects.

In contrast, EVs from mesenchymal stem cells have neuroprotective effects [29,30,31,32].

EVs may therefore induce either pathological or therapeutic effects. To characterize the role of EVs in the mediation of pathological effects from the periphery to the brain in hyperammonemia would allow a better understanding of the mechanisms responsible for MHE and may allow the identification of new therapeutic procedures to prevent or reverse the associated cognitive and motor alterations.

The aim of this work was to assess whether EVs play a role in the induction of glial activation and neuroinflammation in cerebellum and motor incoordination by chronic hyperammonemia. Chronic hyperammonemia in rats induces peripheral inflammation, which mediates the induction of neuroinflammation, leading to alterations in neurotransmission and cognitive impairment. All these effects of hyperammonemia on brain are prevented by inhibiting the induction of peripheral inflammation with anti-TNFα [10]. This rat model is therefore useful to investigate the mechanisms by which peripheral inflammation induces changes in brain and the role of EVs in this process.

To assess if EVs play a role in the mechanisms by which hyperammonemia induces changes in the brain, we isolated EVs from plasma of hyperammonemic rats and assessed whether injection of these EVs to normal rats induces similar changes in neuroinflammation in cerebellum and motor incoordination to those exhibited by hyperammonemic rats.

We have shown that induction of motor incoordination by hyperammonemia is mainly mediated by induction of microglia and astrocytes activation in cerebellum, which is associated to increased TNFα, which activates its receptor TNFR1, leading to increased NF-κB in microglia and expression of glutaminase, which increases glutamate, leading to reversal of the GABA transporter GAT3 function in activated astrocytes and increased GABAergic neurotransmission, finally inducing motor incoordination [9,10,11]. We have therefore analyzed the effects of injecting EVs from hyperammonemic rats to control rats on these mechanisms in cerebellum.

We isolated EVs from plasma of hyperammonemic rats, injected them to control rats and assessed if they transmit the deleterious effects shown in hyperammonemia to the brain. We analyzed the effects on glial activation, neuroinflammation, the changes in TNFα, TNFR1, NF-κB in microglia, glutaminase, GAT3 in cerebellum, and evaluated motor incoordination.

## 2. Materials and Methods

Model of chronic hyperammonemia. Male Wistar rats (6–7 weeks -old) were made hyperammonemic by feeding an ammonium containing diet as described by Felipo et al. [33] during 5 weeks. Rats were sacrificed and plasma was obtained and stored at −80 °C.

Extracellular vesicles isolation. Extracellular vesicles were isolated by ultracentrifugation. Plasma (3 mL) was thawed on ice, diluted to 20 mL with sterile PBS and centrifuged at 2000 g for 20 min at 4 °C. Supernatants were collected, filtered through 0.22 um sterile filters and centrifuged at 150,000× *g* for 16 hours at 4 °C. Supernatants were removed and pellets were washed by ultracentrifugation at 150,000× *g* for 2 hours and 110,000× *g* for 70 min at 4 °C. Pellets were resuspended in 100 μL of PBS and stored at −80 °C. Six microliters were used for protein quantification by the bicinchoninic acid method.

Nanoparticle Tracking Analysis. Distribution profile, size and quantity of vesicles were assessed by Nanoparticle Tracking Analysis with a NanoSight NS300 system (Malvern Panalytical, Malvern, UK) using a 1:100 dilution of exosome samples. Transmission Electron Microscopy. Electron microscopy was performed as described by Thery et al. [34]. Briefly, isolated vesicles were loaded onto carbon-coated copper grids and contrasted with 1% uranyl acetate. Samples were examined with a transmission electron microscope and images were acquired using a digital camera. 

Analysis of protein cargo of EVs by immunoblotting. Samples were subjected to electrophoresis and immunoblotting as described by Felipo et al. [33]. Primary antibodies used were against Alix (1:1000, Abcam), Flotillin-2 (1:500, Fisher), Hsp70 (1:1000, Proteintech), CD9 (1:1000, Abcam), Glutamine Synthetase (1:5000, BD Biosciences), TIMP-3 (1:1000, Abcam), TNFα (1:500, RD systems), TNFR1 (1:500, Abcam), Galectin-3 (1:1000, Abcam). Anti-actin (1:5000, Abcam) was used as protein loading control. Secondary antibodies were anti-rabbit, anti-rat, or anti-goat IgG conjugated with alkaline phosphatase (1:4000, Sigma). Membranes were scanned and band intensities were quantified using Alpha Imager 2200 version 3.1.3.

Proteomic analysis. Differential expression of proteins was analyzed in the SCSIE University of Valencia using EVs isolated from plasma of 8 control and 8 hyperammonemic rats. An equivalent amount of all the samples were pooled to build the spectral library from a 1D SDS-PAGE gel. The run corresponding to the library was cut and digested with sequencing grade trypsin as described by Shevchenko et al. [35]. Five microliters of each digested pool were loaded onto a trap column (NanoLC Column, 3 μm C18-CL, 75 µmx15 cm; Eksigent) and desalted with 0.1% trifluoroacetic acid at 3 μL/min, 5 min. Peptides were loaded onto an analytical column (LC Column, 3 μm C18-CL, 75 µm × 12 cm, Nikkyo) equilibrated in 5% acetonitrile, 0.1% formic acid. Peptide elution was carried out with a linear gradient of 5% to 40% B in 60 min (A: 0.1% formic acid; B: acetonitrile, 0.1% formic acid) for at a flow rate of 300 nL/min. Peptides were analyzed in a mass spectrometer nanoESI qQTOF (5600 TripleTOF, ABSCIEX) and in ProteinPilot search engine (Sciex) to generate peak list. For individual SWATH analysis 20 μg of protein was loaded in a 1D SDS-PAGE gel. Sample was digested with trypsin as above. Peptides were analyzed in the mass spectrometer and the tripleTOF was operated in swath mode, in which a 0.050 s TOF MS scan from 350–1250 *m/z* was performed, followed by 0.080 s product ion scans from 350–1250 *m/z* on the 32 defined windows (3.05 sec/cycle). The Swath windows used were: 15 Da window widths from 450 to 1000 Da, 37 windows. Individual SWATH injections were randomized and analyzed by Peak View 2.1.

**Figure 1:**
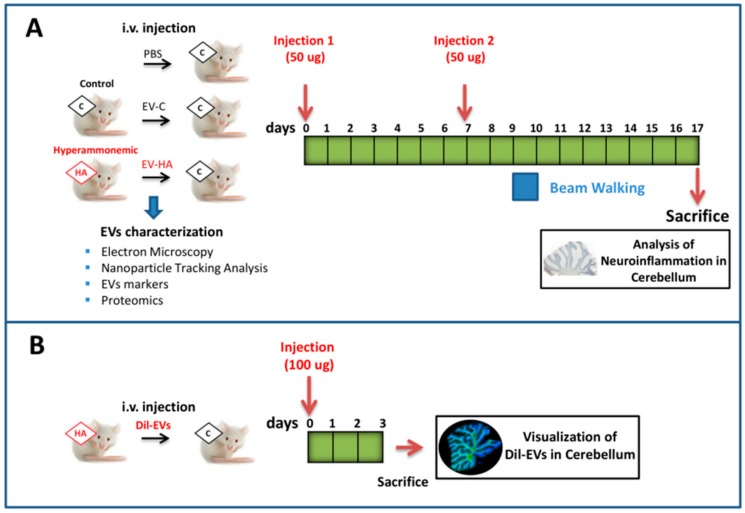
Experimental design. (**A**) Extracellular vesicles from plasma of control and hyperammonemic rats were isolated and characterized by electron microscopy, nanoparticle tracking analysis, extracellular vesicles (EVs) markers, and proteomics. Then, we intravenously injected EVs either from control or hyperammonemic rats (HA) rats into control rats. A third group received only PBS injection. Two injections of 50 µg each were performed on day 0 and day 7. Beam Walking test was performed at day 9 to study motor incoordination. Rats were sacrificed at day 17 and cerebellum was collected for neuroinflammation analysis. (**B**) In a parallel experiment, we injected fluorescently labeled EVs from HA rats into control rats. Rats were sacrificed after 3 days and cerebellum was collected to assess if fluorescent EVs reach this area.

Injection of isolated vesicles to rats. Male Wistar rats (2 months-old, *n* = 22 rats per group) were intravenously injected twice in the tail vein with 50 µg of protein of isolated vesicles from either control or hyperammonemic rats, in 300 µL of sterile PBS. A third group receiving only PBS injection was included as control. The second injection was performed one week later. The experiments were approved by the Comite de Experimentación y Bienestar Animal (CEBA) of our Center and by Conselleria de Agricultura of Generalitat Valenciana and were performed in accordance with guidelines of the Directive of the European Commission (2010/63/EU) for care and management of experimental animals. The experimental design is summarized in Figure 1.

Fluorescent labeling of extracellular vesicles. To check whether injected EVs reach the brain, we performed a parallel experiment in different rats (*n* = 4). Vesicles were labeled with the dye Dil (Sigma) as described by Jiang et al. [36]. Rats were intravenously injected with 100 μg of fluorescently labeled vesicles and sacrificed by perfusion with paraformaldehyde at 72 h. Fixed brains were collected and immediately frozen in OCT. Ten micrometer sections were cut on a cryostat and counterstained with Calbindin (1:500, Abcam), Iba1 (1:300, WAKO), NeuN (1:200, Millipore), GFAP (1:400, Sigma) and Alix (1:200, Abcam), followed by goat anti-rabbit or goat anti-mouse Alexa 488 secondary antibody (1:400, Invitrogen), and DAPI staining. Images were acquired with a Leica TCS SP8 inverted laser scanning confocal microscope using oil objectives: 63X Plan-Apochromat-Lambda Blue 1.4 N.A.

Analysis of protein content in cerebellum. Rats were sacrificed by decapitation and cerebelli of 18 rats per group were dissected and homogenized. Samples were subjected to electrophoresis and immunoblotting as above. Primary antibodies used were against CD68 (1:1000, Abcam), IL-1β (1:500, RD Systems), TNFα (1:500, RD systems), TNFR1 (1:250, Abcam), Glutaminase 1 (1:1000, Novus Biologicals), and GAT-3 (1:1000, Synaptic Systems). Anti-actin (1:5000, Abcam) was used as protein loading control. Secondary antibodies were anti-rabbit, anti-goat or anti-mouse IgG conjugated with alkaline phosphatase (1:4000, Sigma).

Analysis of microglial and astrocytic activation by immunohistochemistry and immunofluorescence. Cerebelli of 4 rats per group were fixed in 4% paraformaldehyde in 0.1 M phosphate buffer for 24 h and embedded in paraffin. Five micrometer sections were cut and mounted on coated slide glass. For immunohistochemistry, sections were sequentially incubated with 3% H2O2 for 15 min to quench endogenous peroxidase activity, blocking serum (normal goat serum) and primary antibodies (4 °C, overnight): Iba1 (1:300, Wako) of GFAP (1:400, Sigma). Then, slides were incubated with ready-to-use Goat anti-rabbit (HRP polymer) or Goat anti-mouse (HRP polymer) secondary antibodies (Abcam) for 1 hour and diaminobenzidine for 10 min. Sections were counterstained with Mayer’s hematoxylin (DAKO) for 5 min. For each rat, 10–12 fields at 56× magnification (Iba1 stained sections) or 40× (GFAP stained sections) were quantified. Images were acquired with Pannoramic Viewer (3DHISTECH). Microglial activation was analyzed by measuring the area of Iba1 stained cells with IpWin 32 software program and astrocytic activation was analyzed by measuring the GFAP stained area with ImageJ software.

For immunofluorescence, sections were incubated with primary antibody against CD68 (1:100, Abcam), rabbit anti-mouse Alexa 488 secondary antibody (1:400, Invitrogen) and DAPI to visualize cell nuclei. For double immunofluorescence, primary antibodies were NF-κB p50 (1:200, Abcam) and Iba1 (1:300, Abcam), followed by donkey anti-mouse Alexa 488 and donkey anti-rabbit Alexa 647 secondary antibodies (1:400, Invitrogen) and DAPI. Eight images per rat were acquired with a confocal microscope using oil objectives: 63X Plan-Apochromat-Lambda Blue 1.4 N.A and the number of CD68 positive cells or number of microglial cells expressing NF-κB was manually counted.

Motor coordination. Beam Walking Test was performed 9 days after the first injection of EVs as described by Gonzalez-Usano et al. [37]. The number of slips committed was recorded.

Statistical analysis. Data are expressed as mean ± SEM. All statistical analyses were performed using GraphPad Prism software 8.1.2 version. Data were analyzed by parametric T-test when comparing two groups or by one-way analysis of variance (ANOVA) followed by Tukey post hoc test when comparing three groups. A confidence level of 95% was accepted as significant.

## 3. Results

### 3.1. Chronic Hyperammonemia Alters the Amount and Protein Cargo of evs in Plasma

**Figure 2:**
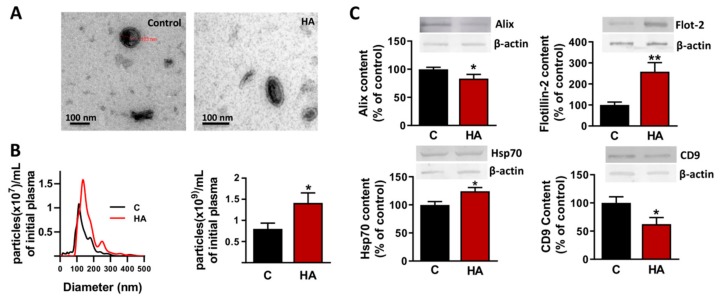
Characterization of extracellular vesicles isolated from plasma of control and hyperammonemic rats. (**A**) Representative images obtained by Transmission Electron Microscopy after negative staining. (**B**) Content of markers Alix (*n* = 16), Flotillin-2 (*n* = 7), Hsp70 (*n* = 20) and CD9 (*n* = 12) analyzed by Western blot. Values are expressed as percentage of protein cargo in control rats and are the mean ± SEM. Data were analyzed by t-test, with values significantly different from control rats indicated by asterisk (* *p* < 0.05,** *p* < 0.01). (**C**) Representative size profile and number of particles obtained by Nanoparticle tracking analysis. Values of particle quantity are the mean ± SEM of 15–18 samples per group. Data were analyzed by t-test, with values significantly different from control indicated by asterisk (*p < 0.01). We assessed if hyperammonemia alters the protein cargo of EVs by performing a proteomic analysis. After Uniprot mammalia library with False Discovery Rate (FDR) identification and analysis of individual SWATH experiments 367 proteins (FDR < 1%) were quantified in the 16 samples. Differential expression analysis identified 124 proteins which content was different in EVs from HA or control rats. 44 proteins (12% of total) were up-regulated, 80 proteins (22% of total) were down-regulated, and 242 proteins (66% of total) remained unaltered (Figure 3A). The differentially expressed proteins identified are listed in Table 1.

We first characterized the EVs isolated from plasma of control and hyperammonemic rats. The EVs exhibited properties compatible with exosomes regarding to morphology, size and surface markers. Electron microscopy images of the isolated EVs are shown in Figure 2A. The presence of surface markers typical of exosomes was evaluated by Western blot, revealing that the isolated EVs were positive for Hsp70, Flotillin-2, and CD9 (Figure 2B). Size distribution profile was analyzed by Nanoparticle Tracking Analysis, showing that the mean diameter of EVs was 143 ± 4 nm (Figure 2C).

The amount of EVs was increased (*p* < 0.05) in hyperammonemic rats to 1.77-fold of control rats (Figure 2C). Different markers of EVs are differentially altered by hyperammonemia. EVs from hyperammonemic rats show increased levels of Flotillin-2 (259 ± 42%; *p* < 0.01) and Hsp70 (124 ± 7%; *p* < 0.05) while the levels of Alix (83 ± 7%; *p* < 0.05) and CD9 (62 ± 11%; *p* < 0.05) are reduced compared to control rats (Figure 2B).

**Figure 3:**
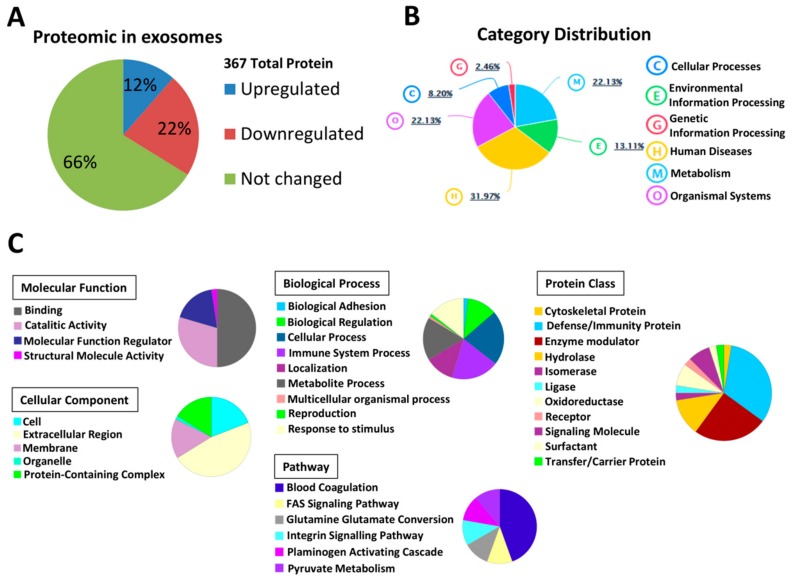
Proteomic profiling of extracellular vesicles purified from plasma and differently expressed in hyperammonemic rats respect to control. (**A**) Number of total proteins identified and percentage of up-regulated and down-regulated proteins, (**B**) Category distribution from Paintomics analysis and (**C**) Gene Ontology (GO) analysis to determine molecular function, cellular components, biological processes, pathways and protein class for the 124 proteins differently expressed in hyperammonemia, plotted with the percentage of each term.

Proteomic category distribution analysis using the PaintOmics 3 application showed that 32% of the proteins are related to human disease, 22% to metabolism and organismal systems, 13% to environmental information processing, 8% to cellular processes, and 2.5% to genetic information processing (Figure 3B).

To gain insight into the potential biological function of proteins differentially expressed we applied Gene Ontology (GO) analysis (Figure 3C). GO annotations are classified in five categories: Cellular component, molecular function, biological process, pathway, and protein class. Significant differences in EVs content were related with proteins activated in the extracellular region and cell surface and with binding and catalytic activity. The differences in EVs proteins are mainly associated with biological processes of immune system, response to stimulus, metabolic processes, glutamine/glutamate conversion, integrin and FAS signaling pathways. Defense/immunity proteins, enzymes modulator and receptors are some protein classes differentially expressed in EVs from hyperammonemic rats (Figure 3C).

**Figure 4:**
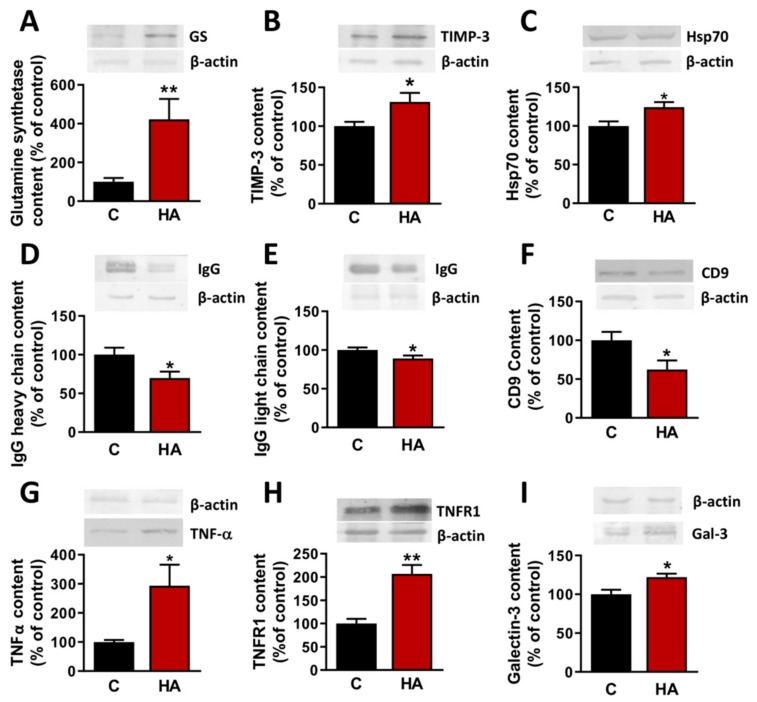
Extracellular vesicles protein cargo analyzed by Western blot. Content of (**A**) glutamine synthetase (*n* = 8), (**B**) TIMP-3 (*n* = 8), (**C**) Hsp70 (*n* = 20), (**D**) IgG heavy chain (*n* = 12), (**E**) IgG light chain (*n* = 16), (**F**) CD9 (*n* = 12), (**G**) TNFα (*n* = 9), (**H**) TNFR1 (*n* = 8–10), (**I**) Galectin-3 (*n* = 7–13). Values are expressed as percentage of protein cargo in control rats and are the mean ± SEM. Data were analyzed by t-test, with values significantly different from control rats indicated by asterisk (* *p* < 0.05,** *p* < 0.01).

We confirmed by Western blot the changes in some of the proteins identified as up-regulated or down-regulated (see Table 1) in hyperammonemia by the proteomic analysis (Figure 4). The contents of glutamine synthetase (Figure 4A), TIMP-3 (Figure 4B) and Hsp70 (Figure 4C) were increased to 422 ± 105% (*p* < 0.01); 131 ± 11% (*p* < 0.05); and 124 ± 6% (*p* < 0.05), respectively, in EVs from hyperammonemic rats compared to controls. The contents of the heavy (Figure 4D) and light (Figure 4E) chains of IgG and of CD9 (Figure 4F) were reduced to 70 ± 8% (*p* < 0.05); 89 ± 4% (*p* < 0.05) and 62 ± 12% (*p* < 0.05), respectively, in EVs from hyperammonemic rats compared to controls.

We also analyzed the content of proteins which could mediate some of the hyperammonemia-induced alterations in cerebellum by Western blot. The content of TNFα (Figure 4G), of the TNFα receptor TNFR1 (Figure 4H) and of galectin-3 (Figure 4I) were increased to 213 ± 50% (*p* < 0.05); 207 ± 19% (*p* < 0.001); and 122 ± 5% (*p* < 0.02), respectively, in EVs from hyperammonemic rats compared to controls.

### 3.2. Injection of EVs from Hyperammonemic Rats Induces Neuroinflammation in Cerebellum in Control Rats

**Figure 5:**
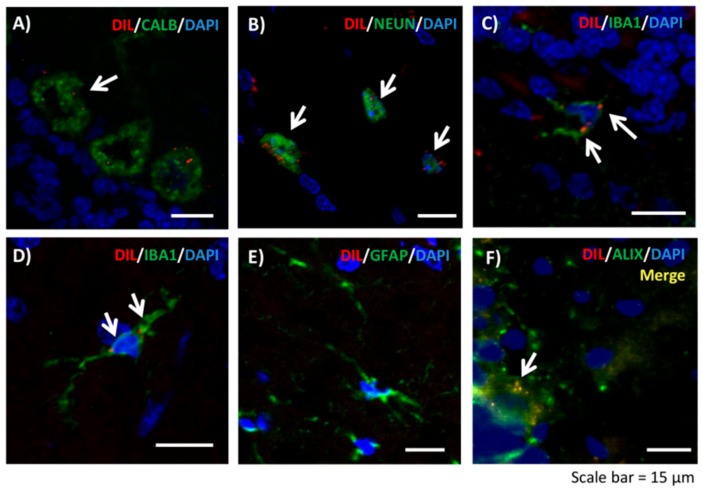
Intravenously injected dil-labeled extracellular vesicles (red) reach the cerebellum after 72 hours. Co-localization was found with (**A**) Purkinje neurons, (**B**) neurons located in white matter, (**C**) microglial cells in the molecular layer, and (**D**) microglial cells in white matter. (**E**) No co-localization was observed with astrocytes in white matter. (**F**) We confirmed that red fluorescence signal co-localizes with Alix, a marker of extracellular vesicles. Scale bar = 15 µm.

We confirmed that injected EVs reached the cerebellum (Figure 5). DIL-labeled EVs (red signal) co-localized with Purkinje neurons (Figure 5A), neurons in white matter (Figure 5B), and microglial cells in molecular layer (Figure 5C) and in white matter (Figure 5D). No co-localization with astrocytes was observed (Figure 5E). The red fluorescence signal of Dil-labeled EVs co-localized with Alix, a marker of EVs, confirming that it corresponds to injected labeled EVs (Figure 5F).

**Figure 6:**
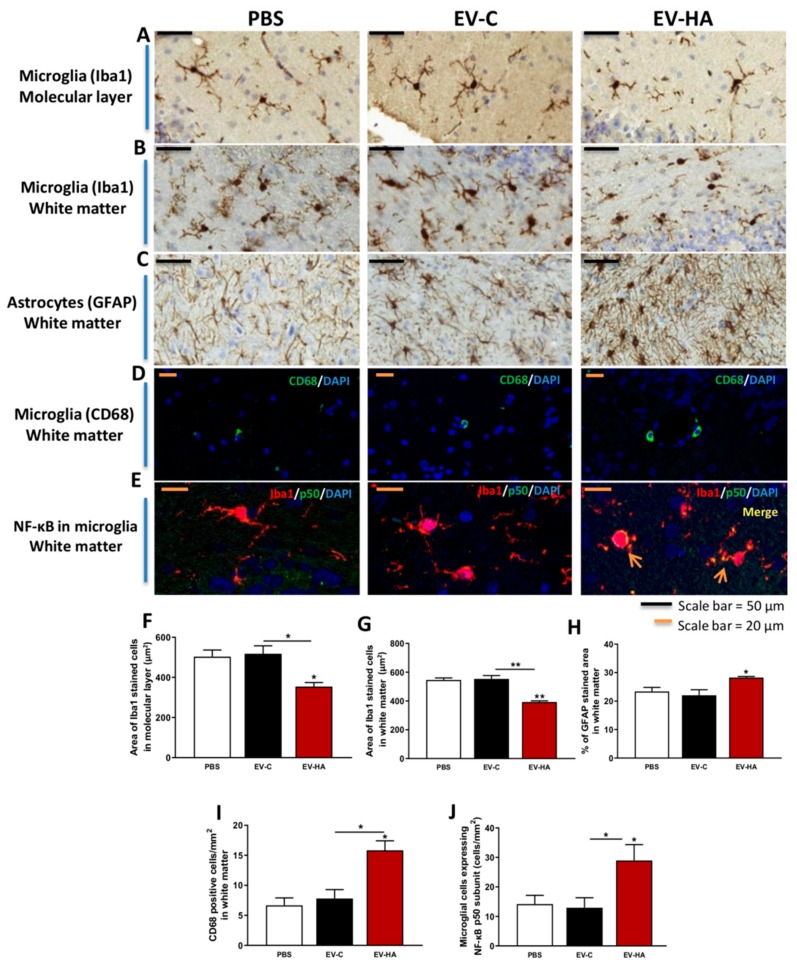
Effects of injected vesicles on microglial and astrocytic activation in cerebellum analyzed by immunohistochemistry/immunofluorescence. Representative images of (**A**) immunohistochemistry against Iba-1 in molecular layer and (**B**) white matter, (**C**) immunohistochemistry against GFAP in white matter, (**D**) immunofluorescence of CD68 in white matter and (**E**) NF-kB p50 subunit expression in microglial cells in white matter. (**F**) Area of microglia (Iba1 stained cells) in molecular layer and (**G**) white matter. (**H**) Percentage of GFAP stained area in white matter. (**I**) Number of activated microglia (CD68 positive cells) in white matter. (**J**) Number of microglial cells expressing NF-kB p50 subunit per mm^2^ in white matter. One-way ANOVA with Tukey post-hoc test was performed to compare all groups. Values are the mean ± SEM of 3-4 rats per group. Values significantly different from PBS-injected rats are indicated by asterisk (* *p* < 0.05,** *p* < 0.01).

We then assessed if injection of EVs from plasma of hyperammonemic rats may transmit deleterious effects to the brain of normal rats. EVs from hyperammonemic rats induced activation of microglia in the molecular layer of cerebellum (Figure 6A), reducing its area (*p* < 0.05) compared to rats injected with PBS (Figure 6F). This effect was specific for EVs from hyperammonemic rats and was not induced by EVs from control rats. Similar microglial activation was observed in white matter (Figure 6B,G). This was further supported by the increased labeling of CD68 (Figure 6D,I), a marker of activated microglia.

EVs from hyperammonemic rats also induced astrocytes activation in white matter (Figure 6C), increasing the GFAP stained area (*p* < 0.05) compared to rats injected with PBS or EVs from control rats (Figure 6H).

**Figure 7:**
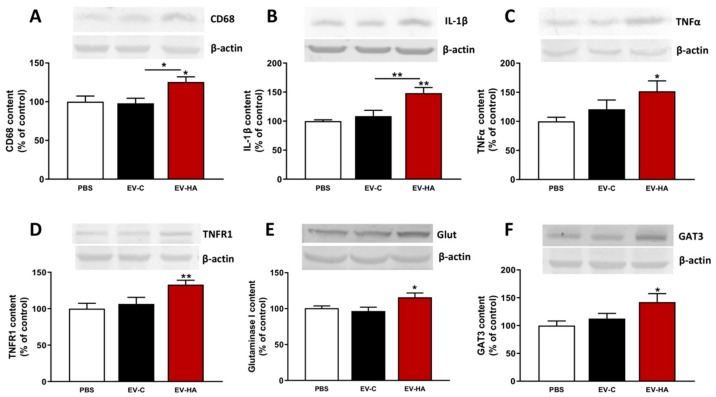
Total content of proteins linked with neuroinflammation in cerebellum of injected rats analyzed by Western blot. Content of (**A**) CD68 (*n* = 9–11), (**B**) IL-1β (*n* = 5–7), (**C**) TNFα (*n* = 9), (**D**) TNFR1 (*n* = 6–11), (**E**) Glutaminase 1 (*n* = 10–13), and (**F**) GAT-3 (*n* = 10–12) in cerebellum homogenates. Representative images of the blots of each protein and the load control (β-actin) are shown. One-way ANOVA with Tukey post-hoc test was performed to compare all groups. Values are expressed as percentage of protein content in PBS-injected rats and are the mean ± SEM. Values significantly different from PBS or EV-C group are indicated by asterisk (* *p* < 0.05,** *p* < 0.01).

We also analyzed the content of pro-inflammatory markers in cerebellum by Western blot. The content of CD68 (Figure 7A), IL-1β (Figure 7B) and TNFα (Figure 7C) was increased to 126 ± 7% (*p* < 0.05); 148 ± 10% (*p* < 0.01); and 152 ± 17% (*p* < 0.05), respectively, in cerebellum of rats injected with EVs from hyperammonemic rats compared to rats injected with PBS or EVs from control rats.

Injection of EVs from hyperammonemic rats induces motor incoordination and the underlying mechanisms in cerebellum in control rats.

Induction of motor incoordination in hyperammonemic rats is mediated by increased content of TNFα in cerebellum, which activates TNFR1, leading to increased NF-κB in microglia and glutaminase, which increases GAT3 in activated astrocytes and GABAergic neurotransmission, leading to motor incoordination [9].

We therefore analyzed the parameters involved in this mechanism in cerebellum of rats injected with EVs from control or hyperammonemic rats. The content of TNFα (Figure 7C), TNFR1 (Figure 7D), glutaminase I (Figure 7E) and GAT3 (Figure 7F) were increased to 152 ± 17% (*p* < 0.05); 133 ± 6% (*p* < 0.01); 116 ± 6% (*p* < 0.05); and 142 ± 15% (*p* < 0.05), respectively, in cerebellum of rats injected with EVs from hyperammonemic rats compared to rats injected with PBS or EVs from control rats.

Injection of EVs from hyperammonemic rats also increased expression of NF-κB in microglia while EVs from control rats did not (Figure 6E,J).

**Figure 8:**
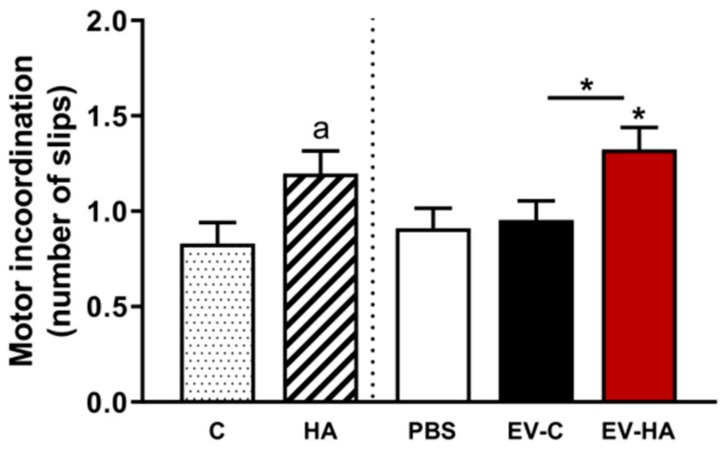
Vesicles isolated from plasma of hyperammonemic rats produce motor incoordination in control rats. Number of slips measured in Beam Walking test. One-way ANOVA with Tukey post-hoc test was performed to compare PBS, EV-C, and EV-HA groups. Values are the mean ± SEM of 19–22 rats per group. Values significantly different from PBS or EV-C group are indicated by asterisk (* *p* < 0.05). Number of slips from control (C) and hyperammonemic rats (HA) without injection are included as reference (*n* = 32–35) and were analyzed by t-test. Values significantly different from control group are indicated by a (*p* < 0.05).

We also assessed the effects of EVs injection on motor coordination using the beam walking test. Injection of EVs from hyperammonemic rats (Figure 8 EV-hyperammonemic rats (HA)) induced motor incoordination, increasing the number of slips to 1.3 ± 0.1 (*p* < 0.05) compared to 0.091 ± 0.1 and 0.095 ± 0.1 in rats injected with PBS (Figure 8 PBS) or EVs from control rats (Figure 8 EV–C), respectively. As a reference, we also analyzed motor coordination in different control and hyperammonemic rats, that showed 0.8 ± 0.1 (Figure 8, C) and 1.2 ± 0.1 (*p* < 0.05) (Figure 8, HA) slips, respectively. These data show that injection of EVs from hyperammonemic rats induces a lack of motor coordination similar to that induced by hyperammonemia per se.

## 4. Discussion

This study provides relevant new insights on the mechanisms by which peripheral changes are transmitted to brain to induce MHE. Understanding these mechanisms in detail may allow designing new, more effective, therapeutic approaches.

This report shows that EVs from plasma of hyperammonemic rats injected to normal rats reach the cerebellum and are enough to trigger the mechanisms that lead to neuroinflammation and motor incoordination in chronic hyperammonemia. This supports the idea that plasma EVs would play a main role in the process by which changes in peripheral inflammation are transmitted to brain to induce neuroinflammation and cognitive and motor impairment in chronic hyperammonemia and MHE.

In contrast, injecting EVs from control rats did not induce any effect on brain. This indicates that differences in the cargo of EVs would be responsible for transmission to brain of the deleterious effects of hyperammonemia.

The analysis of differentially expressed proteins shows that the differences in EVs proteins are mainly associated with biological processes of the immune system, response to stimulus and metabolic processes and that defense/immunity proteins, enzymes modulators and receptors are differentially expressed in EVs from hyperammonemic rats. This suggests that hyperammonemia-induced inflammation play a main role in the changes in the EVs cargo. It is noteworthy that the contents of the pro-inflammatory TNFα and of its receptor TNFR1 are increased 2-fold in EVs in hyperammonemia. As discussed below, these proteins play a main role in the mechanism by which hyperammonemia and MHE induce motor incoordination.

The results reported also show that chronic hyperammonemia increases the number of EVs in plasma. Similar increases have been reported in other pathological situations, mostly associated to inflammation. The amount of EVs is increased in serum of patients with alcoholic hepatitis [38], β-thalassemia [39], endometrial [40] or lung cancer, correlating with tumor stage [41], obesity and diabetes mellitus [42], and chronic obstructive pulmonary disease, associated with systemic inflammation [43] and in serum of children with autism spectrum disorder [26]. Inflammation seems to trigger the increased amount of EVs in most of these situations. The results reported show that hyperammonemia increases the amount and alters the content of plasma EVs. We believe that the effects induced by injection of EVs from hyperammonemic rats to control rats are mediated by the changes in the cargo.

Once injected, EVs reach the cerebellum, in agreement with previous studies showing that EVs can cross the blood–brain barrier [21,25,44]. Into the cerebellum, EVs enter Purkinje neurons, other types of neurons and microglia. The cargo of EVs from hyperammonemic rats would alter the function of these cell types. This results in induction of neuroinflammation, with microglia and astrocytes activation and increased levels of IL-1β and TNFα in cerebellum.

Injection of EVs from hyperammonemic rats also induces motor incoordination, pointing that EVs are enough to induce the neurological alterations associated to hyperammonemia. As we have recently identified the mechanism by which chronic hyperammonemia induces motor incoordination in rats [9], we analyzed whether this mechanism is induced by injection of EVs from hyperammonemic rats and we found that this is the case.

Induction of motor incoordination by chronic hyperammonemia is due to increased TNFα in cerebellum, which activates its receptor TNFR1, leading to increased NF-κB in microglia and glutaminase, which increases GAT3 in activated astrocytes and GABAergic neurotransmission, leading to motor incoordination [9].

We show here that injection of EVs from hyperammonemic rats also induces this mechanism in cerebellum of normal rats, with similar increases in TNFα, TNFR1, and NF-κB in microglia, glutaminase and GAT3. This shows that these plasma EVs carry the molecules necessary to induce this mechanism and motor incoordination in hyperammonemia and MHE. It is noteworthy that EVs from hyperammonemic rats show increased amounts of TNFα and TNFR1, which trigger the mechanism leading to motor incoordination. The specific mechanism by which these proteins transported into the EVs induce the described mechanism leading to motor incoordination cannot be established with the present data and further experiments would be needed to clarify it.

Other proteins enriched in EVs in hyperammonemia may also contribute to this process. For example, galectin-3 is an inflammatory mediator [45] which may be secreted in EVs [46] and is enriched in EVs from hyperammonemic rats. Galectin-3 acts as an endogenous ligand for TLR-4 and induces TLR-4-dependent inflammatory response; its expression is increased in microglial cells activated through various neuroinflammatory stimuli [47]. Galectin-3-dependent TLR4 activation contributes to sustained microglia activation, prolonging the inflammatory response in brain [48]. A role for the galectin-3 transferred into cerebellum by EVs from hyperammonemic rats in the induction of glial activation and neuroinflammation is also possible.

A role for EVs in the transmission of deleterious effects from peripheral blood to brain has been proposed recently in a few studies in other pathological situations and models, mostly associated to sustained inflammation. Ridder et al. [21] purified EVs from blood cells of transgenic mice expressing Cre recombinase specifically in the hematopoietic lineage and injected it into brain of normal mice. These EVs transfer functional RNA to different neuronal types in brain, including Purkinje neurons. This EVs transfer is strongly promoted by peripheral inflammation and induces physiologically relevant changes as indicated by the fact that Purkinje neurons that receive EVs RNA display a different miRNA profile compared to their nonrecombined counterparts. These observations reveal the existence of a mechanism by which EVs communicate signals between the hematopoietic system and brain in response to inflammation.

Peripheral EVs may also trigger neuroinflammation. Li et al. [49] purified serum EVs from a mice model of endotoxemia with LPS and injected them to normal mice. They found that this induced microglial activation, astrogliosis, and increased expression of pro-inflammatory IL-6 and TNFα in hippocampus and cerebral cortex in recipient mice. This supports that during systemic inflammation, circulating EVs may transmit to the brain signals that trigger neuroinflammation.

Intravenous injection to mice of exosomes from serum of patients with Parkinson´s disease evoke symptoms similar to those suffered by the patients, including protein aggregation, dopaminergic neurons degeneration, microglial activation, and movement defects [28].

These reports, together with the present results, suggest that EVs may play a relevant role in the transmission of deleterious effects from peripheral blood to brain in different pathological situations, including hyperammonemia and MHE. There is overwhelming evidence that exosomes, a subtype of EVs, contribute to tumor progression and metastasis [49,50], to the immunopathology in infectious diseases [51] and to neurodegenerative diseases [52]. We show here that EVs also contribute to the induction of neuroinflammation and motor incoordination in hyperammonemia and likely MHE, in which motor incoordination is induced by similar mechanisms.

The cargo of EVs is altered differently in different pathologies and would be responsible for the transmission of the specific pathological mechanisms. The identification of the components of the cargo of EVs responsible for triggering neuroinflammation, the TNFα-TNFR1- NF-κB-glutaminase-GAT3 pathway and motor incoordination in hyperammonemia and of the underlying mechanisms will allow understanding in great detail the pathological events responsible for motor incoordination. This may permit to design more effective treatments to improve motor coordination and function in hyperammonemia and MHE.

## Figures and Tables

**Table 1:** List of up-regulated and down-regulated proteins in extracellular vesicles purified from hyperammonemic rats and obtained by SWATH analysis as indicated in methods.

**Down-Regulated Proteins**			
**Protein Name**	***P*-Value**	**Protein Name**	***P*-Value**
Serine (Or cysteine) peptidase inhibitor, clade C (Antithrombin)	0.011	Pyruvate kinase PKM	0.025
Apolipoprotein H	0.042	Complement component C6	0.005
Cfh protein	0.042	Complement C3	0.015
Carboxypeptidase B2	0.007	Gelsolin	0.008
Hemopexin	0.028	Apolipoprotein A-IV	0.001
Complement component factor h-like 1	0.009	Alpha-2 antiplasmin	0.005
Rat apolipoprotein E protein	0.002	RCG33981, isoform CRA	0.005
Complement C8 gamma chain	0.014	Mannan-binding lectin serine protease 1	0.003
Inter alpha-trypsin inhibitor, heavy chain 4	0.016	Tetraspanin-CD9	0.039
Vitronectin	0.019	Proteasome subunit beta	0.006
Complement factor B	0.017	C9 protein	0.050
Coagulation factor XIII B chain	0.035	Fetub protein	0.013
Complement factor H	0.002	Complement component C8 beta chain	0.017
Retinoic acid receptor responder (Tazarotene induced)	0.010	Serine (Or cysteine) proteinase inhibitor, clade A (Alpha-1 antiproteinase, antitrypsin), member 4	0.002
Prothrombin	0.018	Ig gamma-2B chain C region	0.036
Complement factor I	0.008	Ig gamma-2C chain C region	0.039
Kininogen-1	0.021	Kallikrein B, plasma 1	0.026
MCG1038839	0.002	Complement C4B (Chido blood group)	0.026
Macrophage stimulating 1 (Hepatocyte growth factor-like)	0.005	RCG55135, isoform CRA_b	0.019
Apolipoprotein B-100	0.010	Complement C7	0.048
LOC299567 protein (Fragment)	0.019	Apolipoprotein C-II (Predicted)	0.007
Coagulation factor XII	0.034	Inter-alpha-trypsin inhibitor heavy chain H3	0.039
Complement C8 alpha chain	0.013	MASP-3 protein (Fragment)	0.014
Insulin-like growth factor binding protein complex acid-labile subunit	0.001	Inter-alpha trypsin inhibitor, heavy chain 1	0.033
Carboxypeptidase N catalytic chain	0.013	Inter-alpha-trypsin inhibitor heavy chain 2	0.032
Serine (Or cysteine) peptidase inhibitor, clade C (Antithrombin)	0.011	Peroxidasin	0.014
Apolipoprotein H	0.042	Reelin	0.018
**Up-regulated proteins**			
**Protein Name**	***P*-Value**	**Protein Name**	***P*-Value**
Polymeric immunoglobulin receptor	0.009	Myeloperoxidase	0.011
CD5 antigen-like	0.002	Major urinary protein	0.002
Ig kappa chain V19-17-like	0.042	Brain cDNA, clone MNCb-5810, tissue inhibitor of metalloproteinase 3 (Timp3)	0.010
RCG21066	0.010	Urinary protein 1	0.035
Collectin sub-family member 11	0.039	Ab2-001	0.030
Ceruloplasmin	0.015	Thrombospondin 1	0.005
C4b-binding protein beta chain	0.047	Coagulation factor XI	0.001
Urinary protein 2	0.042	Ficolin (Collagen/fibrinogen domain containing) 1	0.0003
Heat shock protein family A (Hsp70) member 5	0.024	Fc fragment of IgG-binding protein	0.001
Thrombospondin-4	0.004	Galectin-3-binding	0.001
Glutamine synthetase	0.016	Alpha-2-macroglobulin	0.0003
HGF activator	0.031	Ficolin-2	0.002
